# tDCS over left M1 or DLPFC does not improve learning of a bimanual coordination task

**DOI:** 10.1038/srep35739

**Published:** 2016-10-25

**Authors:** Kathleen Vancleef, Raf Meesen, Stephan P. Swinnen, Hakuei Fujiyama

**Affiliations:** 1 KU Leuven, Department of Kinesiology, Movement Control and Neuroplasticity Research Group, 3001 Leuven, Belgium; 2 Newcastle University, Institute of Neuroscience, Framlington Place, NE2 4HH, Newcastle-upon-Tyne, United Kingdom; 3 University Hasselt, Faculty of Medicine and Life Sciences, Rehabilitation Research Center, Agoralaan building D, 3590 Diepenbeek, Belgium; 4 Murdoch University, School of Psychology and Exercise Science, South Street Campus, 90 South Street, Murdoch, Western Australia 6150, Australia

## Abstract

Previously, transcranial direct current stimulation (tDCS) over the primary motor cortex (M1) has resulted in improved performance in simple motor tasks. For a complex bimanual movement, studies using functional magnetic resonance imaging and transcranial magnetic stimulation indicated the involvement of the left dorsolateral prefrontal cortex (DLPFC) as well as left M1. Here we investigated the relative effect of up-regulating the cortical function in left DLPFC and left M1 with tDCS. Participants practised a complex bimanual task over four days while receiving either of five stimulation protocols: anodal tDCS applied over M1, anodal tDCS over DLPFC, sham tDCS over M1, sham tDCS over DLPFC, or no stimulation. Performance was measured at the start and end of each training day to make a distinction between acquisition and consolidation. Although task performance improved over days, no significant difference between stimulation protocols was observed, suggesting that anodal tDCS had little effect on learning the bimanual task regardless of the stimulation sites and learning phase (acquisition or consolidation). Interestingly, cognitive performance as well as corticomotor excitability did not change following stimulation. Accordingly, we found no evidence for behavioural or neurophysiological changes following tDCS over left M1 or left DLPFC in learning a complex bimanual task.

Over the last decade, the effect of transcranial direct current stimulation (tDCS) on human motor performance and learning has been extensively investigated. A number of studies applying tDCS on the primary motor cortex (M1) have shown positive effects on motor task performance and learning^1^ in both healthy^2^,^3^,^4^ and clinical populations, such as stroke patients^5^,^6^. This has been related to increased excitability of M1 as evaluated by transcranial magnetic stimulation (TMS)^2^,^3^.

While a variety of motor tasks have been shown to benefit from the application of tDCS, these tasks are predominantly simple motor tasks performed with a single effector^3^,^7^,^8^,^9^,^10^,^11^,^12^,^13^. Despite the abundance of research on the effect of tDCS on unimanual motor tasks, only a limited number of studies have investigated the effect of tDCS on more complex tasks such as bimanual coordination^14^. The ability to perform bimanual movement in a coordinated manner is essential for our daily activity since many everyday tasks (e.g., tying shoelaces) require coordinated motion of two hands in time and space.

A growing body of research has highlighted that a broader set of key brain regions are functionally involved in the execution of bimanual coordination^15^,^16^. A recent functional magnetic resonance imaging (fMRI) study from our group revealed the involvement of prefrontal brain regions, including bilateral dorsolateral prefrontal cortex (DLPFC), in addition to M1, during a complex bimanual coordination task^17^. Left DLPFC activations were particularly evident during the planning phase when a visual cue informed the subject about the forthcoming required movement. Left M1 activation during planning and execution of the movement was reduced after training, suggesting the pivotal role that left M1 plays in skill acquisition. In line with this finding, a recent study using repetitive transcranial magnetic stimulation (rTMS) demonstrated that the increased cortical excitability in the left M1 was associated with superior learning in a bimanual task^18^. Similarly, our recent transcranial magnetic stimulation (TMS) studies provided a strong support to this view by revealing the task-specific modulations in interhemispheric projections from DLPFC to contralateral M1^19^,^20^. A key function of the DLPFC is thought to integrate information maintained in working memory with the organization of upcoming actions^21^,^22^. Furthermore, the left DLPFC is engaged in encoding, while the right DLPFC is engaged in retrieval of information^23^. Although the increased activation in the DLPFC during the planning period of a bimanual coordination task fits nicely with the idea that the DLPFC links information (i.e., a visual cue) to the regulation of a forthcoming action (i.e., required movement), whether the DLPFC is actively regulating the bimanual movement (i.e., causality) is still elusive. Importantly, the effect of tDCS over DLPFC has only been investigated in the context of cognitive tasks (e.g., working memory and executive control task)^24^,^25^,^26^,^27^,^28^,^29^, but the influence of enhanced DLPFC function on motor behaviour is largely unknown. Furthermore, effects of tDCS over M1 are mostly demonstrated in simple tasks, few studies have looked into more complex motor tasks^9^,^30^,^31^. One example of a study investigating a more complex motor task is by Furuya and colleagues showing thatM1 stimulation only increases performance in novice piano players but not in professional players^14^.

Moreover, previous research has shown differential effects for two components of the learning process: acquisition and consolidation. During acquisition, encoding of information takes place and improves performance, while learning can only take place following consolidation^32^. For the acquisition phase, a previous fMRI study showed decreased activations in the bilateral DLPFC after five days of training of a bimanual movement task^17^, suggesting that DLPFC is primarily involved in the initial acquisition phase. Instead, there is evidence to suggest that the primary motor cortex (M1) is highly involved in both acquisition^33^ and consolidation^34^. Furthermore, the effect of tDCS on motor task performance is highly task-dependent^35^,^36^. For example, greater gains in both acquisition and consolidation following M1 stimulation was observed in a serial reaction time task^8^. While, in a more complex sequential force control task, M1 stimulation only improved consolidation and not acquisition of the new skill^9^.

In the current study, we aim to up-regulate the function of left DLPFC and left M1 with tDCS to enhance the learning of a complex bimanual coordination task while considering the two phases of learning, i.e., acquisition and consolidation. For this purpose participants were randomly assigned to one of five experimental groups: (1) anodal tDCS over left DLPFC (aDLPFC), (2) sham tDCS over left DLPFC (sDLPFC), (3) anodal tDCS over left M1 (aM1), (4) sham tDCS over left M1 (sM1), and (5) behavioural tasks without tDCS (BEHAV). This design allowed us to identify a potential placebo effect in the sham tDCS groups. To enable direct comparison with the studies described above^17^,^19^,^20^,^37^, we utilised the established bimanual tracking task (BTT) in the current study. The goal of the BTT was to accurately track a moving target presented on a screen by rotating dials^17^,^37^,^38^. The task enables us to systematically manipulate task difficulty by training subjects in various ratios of rotation frequency between the left and right hand: (a) an easy isofrequency ratio in which both hands rotate in the same frequency, and (b) more difficult multifrequency ratios in which one hand rotates faster than the other hand. Due to the complex nature of the task and earlier recommendations for multiple tDCS sessions over a single session^29^,^39^,^40^ we trained our participants over multiple days (Day 1–4) with a retention test which was performed 7 days after the last day of training (Day 11). Furthermore, the multi day training is particularly useful to disentangle the effect of tDCS on two components of the learning process (i.e., acquisition and consolidation). Accordingly, skill acquisition was quantified measuring skill changes during the session, while consolidation was quantified obtaining skill changes between sessions (i.e., post-session to subsequent pre-session scores). In addition, excitability changes after left M1 stimulation were evaluated. Also subjective experiences of stimulation and potential confounding variables like sleep and caffeine consumption were questioned in daily questionnaires to account for the effects of non-experimental variables.

To this end, we hypothesized that the effect of tDCS over left DLPFC and left M1 on bimanual performance would depend on the task difficulty and the phase of learning. Specifically, we predicted that particularly the difficult task conditions would benefit from left DLPFC stimulation during acquisition as previous studies using our bimanual tracking task (BTT) showed extensive involvement of left DLPFC during this phase^17^,^19^,^20^ and more cognitive control is required in difficult task conditions. In contrast, left M1 stimulation would improve the performance in easy conditions in both skill acquisition and consolidation, but improvement of more difficult conditions would be only evident in the consolidation phase.

## Results

### BTT

Performance on the bimanual tracking task was measured by the average target deviation during each trial. Only trials of the daily pre- and post-training measurements were included in analyses since performance on these trials are not mediated by feedback. Missing data of two independent sessions (one pre-measurement on day 2 and one pre-measurement on day 4 in two different subjects) due to technical problems were replaced by the mean target deviation of the other subjects belonging to the same group separately for each frequency ratio. BTT data were normalized to each individual baseline by dividing the target deviation of each trial by the average target deviation of the first session (pre-measurement on Day 1). To meet the assumption of normality, a log transformation was applied.

**Figure 1:**
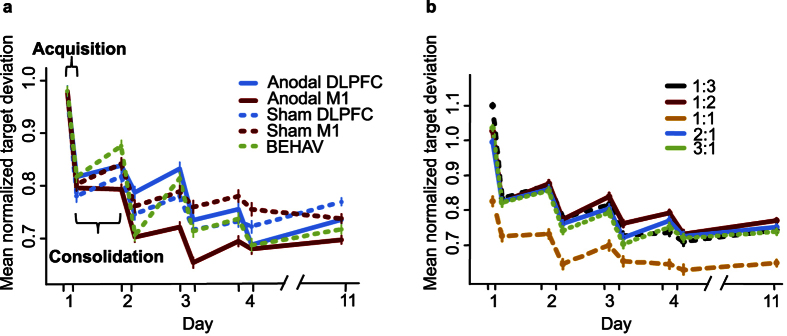
BTT results. The averaged and normalized target deviation is plotted for all pre- and post-measurements on each day. The first dot on each day (x-axis) represents the pre-measurement, the second dot represents the post-measurement. Error bars indicate 1 standard error above and below the mean. In (**a**) differences between stimulation groups are shown. In (**b**) learning curves for each frequency ratio are plotted.

To study *general effects* of tDCS on motor learning, we performed a mixed factorial ANOVA with factors Group (active M1, sham M1, active DLPFC, sham DLPFC, no tDCS), Time (pre- and post-measurement Day 1–4, 11), difficulty level as reflected in Frequency Ratio (1:1, 1:2, 1:3, 2:1, 3:1) and all interactions. We observed a main effect of Time (F(5.38, 376.94) = 32.15, p < 0.001, BF_10_ = 3.20 × 10^151^), indicating a general reduction of average target deviation over days of training. Also the main effect of Frequency Ratio (FR) was significant (F(2.37, 165.70) = 34.67, p < 0.001, BF_10_ = 1.05 × 10^155^). Planned comparisons showed significant differences between isofrequency (FR 1:1) and multifrequency (FR 1:2, 2:1, 1:3, 3:1) trials (all p < 0.001) and not between multifrequency trials (0.05 < p < 0.84). As can be seen in Fig. 1B, performance is better in isofrequency trials (yellow dotted line in Fig. 1B) compared to multifrequency trials. No evidence for a main effect of Group was observed (F(4, 70) = 1.00, p = 0.41, BF_10_ = 0.04), indicating no difference between stimulation groups over all time points. However, the interaction between Group and Time was significant (F(21.54, 376.94) = 1.77, p = 0.02). Figure 1A suggests that subjects who received anodal stimulation over M1 showed a reduced average target deviation during the third day of training, and this difference seems to disappear on the fourth day of training. However, post hoc pairwise comparisons between Groups on post-performance on Day 3 and pre-performance on Day 4 indicate the differences between Groups are not significant following Bonferroni-Holm correction (0.26 < p < 1) The interactions between Time and FR (F(15.37, 1076.05) = 1.38, p = 0.15), Group and FR (F(9.47, 165.7) = 1.24, p = 0.27) and the tree-way interaction (F(61.49, 1076.05) = 1.09, p = 0.30) did not reach significance level. A JZS Bayes factor repeated measures ANOVA^41^ with default prior scales revealed that the main effects model with factors FR and Time was preferred to the main effects model with factors FR, Time, and Group by a Bayes factor of 0.05 and to the interaction model (FR, Time, and FRxTime) by a Bayes factor of 7.40 × 10^−7^. With respect to the first, this means that the data are 1/0.05 or 20 times more likely to occur under a model without-an effect of Group, rather than a model with it. In other words, the data provide strong evidence against the hypothesis that Group influences BTT performance according to the criteria by Jeffreys^42^.

Next we examined the effect of tDCS on *acquisition* by performing a mixed factorial ANOVA on the difference between post- and pre-performance on each day (e.g. post-measurement Day 2 – pre-measurement Day 2) with factors Group, Time and FR. During acquisition, performance change was most pronounced at the first day and reduced over days (main effect of Time: F(2.64, 184.62) = 29.83, p < 0.001, BF_10_ = 4.27 × 10^35^). No significant difference between stimulation groups was observed (F(4,70) = 1.17, p = 0.33, BF_10_ = 0.02). In addition, we found evidence for a main effect of FR (F(2.56, 179.55) = 8.22, p < 0.001, BF_10_ = 18.47), with a stronger reduction in target deviation in the multifrequency trials than in the isofrequency trials (all p < 0.01) and no difference between multifrequency trials (0.06 < p < 1). The difference between FRs decreased over days as indicated by a significant interaction between Time and FR (F(8.12, 568.70) = 3.18, p = 0.001). Importantly, interactions including Group as a factor were not significant (Group × Time: F(10.55, 184.62) = 1.45, p = 0.16; Group × FR: F(10.26, 179.55) = 1.18, p = 0.31; Group × Time × FR: F(32.5, 568.7) = 0.89, p = 0.64), indicating similar changes in performance were achieved by all the stimulation groups during acquisition. The main effects model with factors FR and Time was preferred to the main effects model with factors FR, Time, and Group by a Bayes factor of 0.03 and to the interaction model (FR, Time, and FRxTime) by a Bayes factor of 0.88. The data provide very strong evidence in favour of the hypothesis of no effect of Group on acquisition of BTT (1/0.88 or 33.33 times more likely).

The effect of *consolidation* was assessed by calculating differences between pre- and post-measurements of consecutive days (e.g. pre-measurement Day 2 – post-measurement Day 1) and we submitted the result as a dependent variable to a mixed factorial ANOVA with factors Group, Time and FR. Changes in target deviation during the consolidation interval did not differ significantly between groups (F(4,70) = 1.24, p = 0.30, BF_10_ = 0.03), between days (F(2.91, 203.82) = 1.23, p = 0.30, BF_10_ = 0.06), and between frequency ratios (F(2.92, 204.52) = 2.02, p = 0.11, BF_10_ = 0.02). Neither did we observe any significant interactions (Group × Time: F(11.65, 203.82) = 1.23, p = 0.26; Group × FR: F(11.69, 204.52) = 1.63, p = 0.09; Time × FR: F(8.69, 608.03) = 1.06, p = 0.39; Group × Time × FR: F(34.74, 608.03) = 0.93, p = 0.59). In addition, no evidence for a difference between stimulation groups was found when looking at the first consolidation interval only (F(4, 70) = 1.15, p = 0.34, BF_10_ = 0.11). The Bayes factors indicate that the data provide very strong evidence against the hypothesis that Group, Time or FR influences consolidation of BTT. In sum, these results suggested that the application of anodal tDCS did not have an additive effect on learning during consolidation phases, regardless of the stimulation site.

### Motor evoked potential (MEP)

To investigate the effect of tDCS on corticospinal excitability, we obtained MEP amplitudes in the right flexor carpi radialis (FCR) muscle before and after the training sessions in each session from subjects receiving anodal tDCS over left M1 (aM1) and sham tDCS over left M1 (sM1).

**Figure 2:**
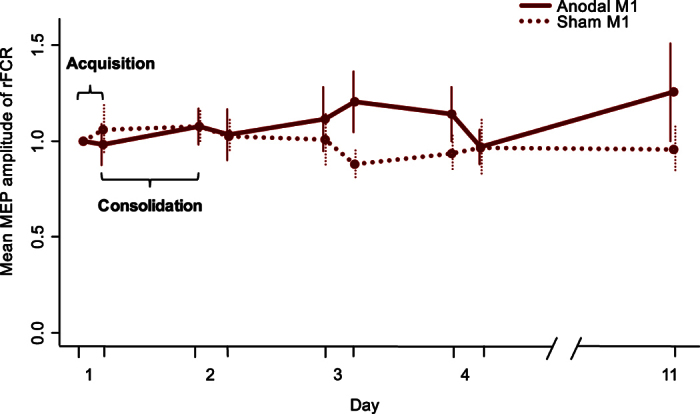
MEP results. The averaged and normalized MEP amplitude of right FCR is plotted for all pre- and post-measurement on each day. The first dot on each day (x-axis) represents the pre-measurement, the second dot represents the post-measurement. Error bars indicate 1 standard error above and below the mean. Acquisition data can be read from this graph by comparing the difference between the pre- and post-measurement of each day. Consolidation data can be read by comparing the difference between the post- and pre-measurement of two consecutive days.

To evaluate the overall effects of tDCS over days, a mixed factorial ANOVA with normalized mean MEP amplitude as the dependent variable and with factors Group (anodal M1 stimulation versus sham M1 stimulation) and Time (post-measurement of Day 1, pre- and post-measurement Day 2–3, and post-measurement 11) was performed. There was no significant main effect of Group (F(1, 28) = 0.70, p = 0.41, BF_10_ = 0.35), neither did we observe a main effect of Time (F(3.79, 67.67) = 0.27, p = 0.81, BF_10_ = 0.03) or an interaction between Group and Time (F(3.79, 67.67) = 1.04, p = 0.37) (Fig. 2). JZS Bayes factor repeated measures ANOVA revealed that the null effects model with a random effect of subjects was 33.33 (or 1/0.03) and 2.86 (or 1/0.35) more likely than main effect models with factors Time or Group respectively.

No significant effect of Group on MEP amplitude change *during acquisition* (by subtracting MEP amplitude obtained prior to the tDCS from MEP amplitude obtained after the tDCS) was found with a mixed factorial ANOVA (F(1, 28) = 0.02, p = 0.89, BF_10_ = 0.27). In addition, no evidence for a main effect of Time was observed (F(2.79, 78.01) = 0.27, p = 0.83, BF_10_ = 0.06), neither for the interaction of Time and Group (F(2.79, 78.01) = 1.37, p = 0.26).

Similarly, no Group differences were observed in MEP amplitude change *during the consolidation interval* (by subtracting pre-MEP amplitude from the post-MEP amplitude of the previous day) (F(1,28) = 0.41, p = 0.53, BF_10_ = 0.31), which did not vary over Time (F(2.46, 68.78) = 0.36, p = 0.74, BF_10_ = 0.07). Neither did we observe evidence for an interaction between Time and Group (F(2.46,68.78) = 0.73, p = 0.51). Overall, these results suggested that changes in corticospinal excitability following anodal tDCS was comparable with sham stimulation of left M1 and did not change, even after the simultaneous training and tDCS.

Last, a Two Step cluster analysis on the ratios between pre- and post-MEP measurements of Day 1 was conducted^43^,^44^. This analysis could not convincingly distinguish responders and non-responders. When data of the aM1 and sM1 group were submitted in one analysis, two clusters were identified but subjects of both stimulation groups, i.e., anodal and sham stimulation, were equally distributed over both clusters. A Two Step cluster analysis on data of the anodal group only resulted in one uniform cluster.

### Working memory

Details of inferential statistics can be found in Supplementary Table S1. Data in the working memory tasks were analysed by mixed factorial ANOVAs. In the Go/No-Go task, sustained attention did not significantly differ between subjects receiving anodal tDCS over left DLPFC (aDLPFC), sham stimulation over left DLPFC (sDLPFC), and no stimulation (BEHAV) (p = 0.39, BF_10_ = 0.36), and a significant interaction between Group and Day was not observed (p = 0.39). In set shifting, a main effect of Group showed an approaching level significance (p = 0.05, BF_10_ = 1.71). However, unlike our expectation, this was driven by the better performance in both stimulation groups (aDLPFC, 81.5% and sDLPFC, 83.4%) compared to the BEHAV group (75.4%). For response inhibition, no significant effects of Group (p = 0.94, BF_10_ = 0.33) or Day by Group (p = 0.46) were found. In the 3-back task, no significant effects of Group and Group by Day were observed in percentage correct (Group: p = 0.83, BF_10_ = 0.33; Group × Day: p = 0.57), percentage false positives (Group: p = 0.79, BF_10_ = 0.34; Group × Day: p = 0.81), and reaction time (Group: p = 0.35, BF_10_ = 0.33; Group × Day: p = 0.46). These results indicate no performance difference between anodal, sham and no tDCS over left DLPFC in any of the working memory tasks.

### Questionnaires

Only main findings are described here and additional details on data-analyses are reported in Supplementary Table S2. A one-way ANOVA indicated no differences between Groups in sleep quality (p = 0.24, BF_10_ = 0.12), sleep duration (p = 0.27, BF_10_ = 0.20), alcohol intake (p = 0.58, BF_10_ = 0.09) or caffeine intake (p = 0.76, BF_10_ = 0.11) that could have confounded our results. We observed no significant difference in tDCS sensations between anodal and sham tDCS groups (p = 0.71, BF_10_ = 0.37). However, a main effect of Group was observed for the starting time of sensations, with subjects reporting a later starting time in the sham tDCS groups (p = 0.02, BF_10_ = 2.91). The main effect of Group did not reach significance level for the duration of sensations (p = 0.13, BF_10_ = 0.74) and the subjective influence of sensations on performance (p = 0.12, BF_10_ = 0.78).

## Discussion

Both at the behavioural level and the neurophysiological level, we did not observe evidence for an effect of tDCS over left DLPFC or left M1 during acquisition or consolidation of a complex bimanual task, independent of task difficulty.

We observed an overall learning effect on BTT performance (increased performance over training days). Performance was better in iso-frequency ratios compared to multi-frequency ratios. Our results correspond to earlier findings with this task^17^,^37^,^38^. However, we were the first to look into the specific effect of acquisition and consolidation across multiple training days, providing higher resolution to the evolution of learning a bimanual coordination task. While performance change in the bimanual skill was most pronounced during acquisition on the first training day, during consolidation performance changes were similar on each day. This improvement in performance during acquisition, but without an additive effect of consolidation during sleep, is in accordance with a study by Waters-Metenier, Husain, Wiestler, and Diedrichsen^36^. Waters-Metenier and colleagues observed that configuration learning (a recurrent production of difficult hand muscle activation patterns) was improved after acquisition, but not following a consolidation interval. Furthermore, in the acquisition phase in our study, the improvement in performance over training days is less pronounced in iso-frequency ratios than in multi-frequency ratio since iso-frequency ratios offer less room for improvement. Consequently, differences in performance between frequency ratios are reduced at the end of training. We did not observe differences between frequency ratios in consolidation.

Previous studies from our research group demonstrated that the bilateral DLPFC was highly involved in the acquisition of the same complex bimanual task^17^,^19^,^20^, however the current study shows that left DLPFC stimulation does not result in performance improvement, neither during acquisition in difficult task conditions as was predicted, nor during consolidation and in easy task conditions. In working memory tasks, both positive behavioural effects^24^,^26^,^27^,^29^ and null effects^28^,^45^,^46^ of tDCS stimulation over DLPFC have been reported. A review^25^ concluded that performance in an n-back is not influenced by tDCS, whereas reaction time performance benefits from tDCS over DLPFC. More recently, Horvath, Forte, and Carter^47^ conducted a meta-analysis on the cognitive effects of tDCS and concluded there was no evidence for improved performance in an n-back task following a single session of stimulation over the left DLPFC^48^. They suggested a beneficial effect of tDCS might be found after multi-day sessions. However, our results indicate that stimulation over left DLPFC during four consecutive days likewise did not improve performance on working memory task. The lack of improvement in cognitive tasks does not necessarily imply that DLPFC is not involved in the task, rather, the increased cortical excitability by tDCS may not directly translate to improved functioning.

No overall differences in BTT performance were observed between the stimulation groups. Although the aM1 group showed higher performance after Day 3 relative to the other groups, this difference was not significant. We did not observe any evidence that anodal stimulation of left M1 specifically influenced acquisition or consolidation of learning a complex bimanual movement. Neither did we observe differences in the effect of left M1 stimulation between task difficulty levels. Similarly, at the neural level, we observed no effect of anodal tDCS over left M1 on corticospinal excitability measured with MEP amplitude. Our questionnaire results indicate that this cannot be related to an ineffective sham condition or sleep, alcohol, or caffeine consumption. This is in contrast to previous studies that have shown an increase in corticospinal excitability and behavioural performance in simple tasks after M1 stimulation^3^. However, more recently, the prevalence of substantial interindividual differences and dissimilar results for patients, elderly and healthy controls have questioned the generalizability of neurophysiological changes following tDCS. In addition, uncertainties about the mechanisms underlying tDCS, result in the lack of a common protocol that may increase variability in tDCS results. In the following paragraphs, we discuss how several of these factors have potentially influenced our findings.

Potentially, an explanation can be found in the tDCS protocol that we used. First, one could argue that applying tDCS during the task (online tDCS) instead of before the task (offline tDCS) has caused interference and abolished potential effects^49^. However, other authors have argued for delivering stimulation during learning of a motor task^50^. Empirical evidence has provided strong support for the effects of tDCS both during^8^,^23^,^40^,^51^ and before a behavioural task^45^,^46^,^52^,^53^,^54^, suggesting that online or offline stimulation protocols do not give different results. These results were further confirmed by a meta-analysis^25^. As such, we consider that the timing of stimulation (online) is an unlikely explanation for not observing an effect of tDCS.

Second, it is unlikely that ineffective blinding has confounded our results. We did not observe a significant difference in reported sensations following active and sham stimulation, which indicates subjects were blind to the stimulation condition. In addition, we tested whether a placebo effect of tDCS had obscured a difference between anodal and sham stimulation by including a behavioural group who received no stimulation. Because performance did not differ between the groups who received sham stimulation (sM1 and sDLPFC) and the behavioural group (BEHAV), a placebo effect explaining the lack of differences between groups is also unlikely.

Third, we tried to reduce intra-subject variability due to the effects of circadian and metabolic rhythms by planning training sessions at the same time of the day at the four consecutive training days. Cluster analysis showed no indication for clusters of responders and non-responders in our MEP data.

Fourth, potentially the cathode has induced stimulation in the right frontal cortex. However, to reduce the influence on brain areas under the cathode, we have used a cathodal electrode of 6 × 8.5 cm which is larger than the conventional size of 5 × 5 cm. Previously, it has been shown that increasing the size of the electrode turns the cathode effectively in an inactive electrode^55^. This manipulation does not eliminate the influence of the cathode: the less effective cathode might still have reduced the current density and made the stimulation less effective.

Besides the tDCS protocol, one might argue that our healthy young subjects reached ceiling performance in the BTT, which eliminated room for improvement with tDCS. Therefore, stronger effects might have been found by testing and elderly population as there is some evidence to suggest that older adults have greater responsiveness to tDCS^56^,^57^. Besides elderly, patient populations might benefit more from tDCS than healthy subjects^25^. In addition, stimulating the non-dominant hemisphere instead of the relatively overexcited left hemisphere might enhance the effect of tDCS^8^,^36^,^40^. However, a previous study showed that the BOLD signal from left M1 decreased during training^17^, suggesting that the initial increased activation in left M1 was functional. Despite these findings, a ceiling effect in our young healthy sample is an unlikely explanation for our results. Although this explanation may hold for the last training day, on the earlier days subjects clearly did not reach their optimal performance level.

A more likely explanation for the limited effect of tDCS can be sought in network interactions. A recent TMS study from our group^20^ showed that in order to successfully perform this complex bimanual movement, interactions between left PMd and right M1 need to be regulated in a task-specific manner by flexibly modulating the amount of facilitatory and inhibitory interactions^58^. Therefore, the modulation of a single cortical region may not be optimum to enhance the task performance and acquisition for BTT. Similarly, because BTT requires multiple forearm muscles to contract in a timely manner at different timing, measuring MEP changes in a single muscle (FCR) might not be optimal to observe changes in the corticospinal projection associated with learning the complex motor task. Therefore, assessing (by TMS) FCR representation in left M1 might have not be sensitive to capture the plastic changes associated with the BTT training (see also ref. 59), suggesting that changes in cortical excitability are not related to changes in motor performance. In this regard, transcranial alternative current stimulation (tACS) may be useful. tACS utilises a time-varying current stimulation, alternating between electrodes. The application of tACS can modulate the power of oscillatory rhythms in the cortical areas in a frequency-dependent manner by synchronizing or desynchronizing neuronal networks. Therefore, tACS may be able to improve BTT task performance inducing the network-intrinsic frequency modulation and inter-regional coupling within the motor network intrinsic for the complex bimanual movements.

In sum, we investigated the effects of tDCS over left M1 and left DLPFC on learning of a complex bimanual task. We did not observe any additive learning effects of tDCS in acquisition or consolidation processes regardless of the task difficulty. Based on these results, we conclude that tDCS over left M1 and DLPFC has no beneficial effect on bimanual performance, at least with the parameters used and for the subjects sample tested in the current study. The potential reason is that the stimulation did not alter underlying mechanisms of bimanual performance including corticomotor system (following M1 stimulation) or cognitive function (following DLPFC stimulation). As such, we suggest that the effect of tDCS on behaviour may be limited to certain tasks and cohorts.

## Methods

### Participants

Seventy-five university students participated in the study with an age range between 19 and 30 (47 females and 28 males). Subjects were randomly assigned to one of five experimental groups, with 15 subjects in each group. No significant differences were observed between experimental groups in age or handedness (see Supplementary Table S2). A screening for contra-indications of tDCS and TMS was administered prior to participation. Participants gave their written informed consent and all procedures were approved by the Ethical Committee of the University of Leuven (KU Leuven) and were in accordance with the Declaration of Helsinki (1964).

### Design and procedure

**Figure 3:**
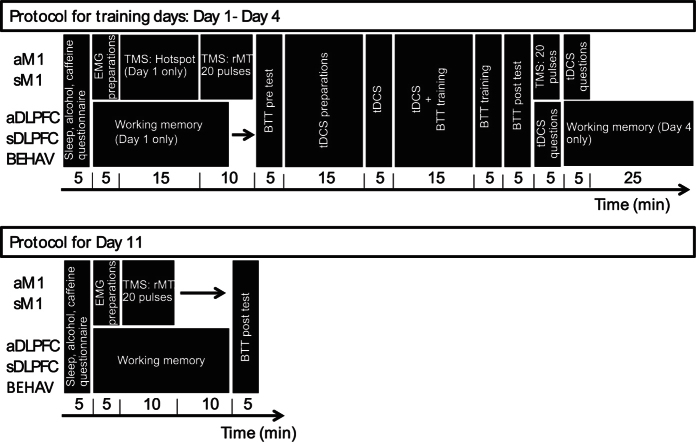
Schematic overview of the protocol. The top panel shows the protocol for the training days or all stimulation groups. The bottom panel shows the protocol for the retention day. All blocks followed immediately after each other as indicated by the arrows.

A schematic representation of the main procedure is given in Fig. 3. In four daily sessions (Day 1–4), subjects were trained in a bimanual tracking task. For each subject, all the sessions were conducted at a similar time of the day (±2 h). Prior to each training session, a questionnaire assessed the quality and quantity of sleep, caffeine and alcohol intake to evaluate differences in these confounding variables between the experimental groups. In addition, every day a pre-measurement of the bimanual tracking task was applied. After the pre-measurement, tDCS was applied for 20 minutes. After five minutes of tDCS the bimanual tracking training session of 20 minutes was started. The stimulation was continued for 15 minutes during the training. Following the training, a post-measurements of the bimanual tracking task was administered. Furthermore, a tDCS sensation questionnaire was administered to evaluate effectiveness of blinding subjects to the stimulation type (sham versus anodal tDCS). Six to seven days after the last training session (~Day 11), a delayed post-measurement of the bimanual tracking task was completed without any tDCS to assess long-term consolidation effects.

We assessed working memory in the groups aDLPFC, sDLPFC and BEHAV to assess earlier findings on the effect of DLPFC stimulation on working memory. Two well-known working memory tasks were administered: a parametric Go/No-Go task and a 3-back task. To assess the short and long term effects of left DLPFC stimulation, subjects were assessed before the first tDCS session (pre-test on Day 1), after the last tDCS session (post-test on Day 4) and during the delayed post-measurement, one week later (Day 11).

For the group who received stimulation over left M1 (sM1 and aM1), we assessed changes in corticospinal excitability using TMS. Corticospinal excitability was examined immediately before and following tDCS (i.e., anodal and sham on Days 1 through 4) by applying single-pulse TMS to the left M1 targeting right flexor carpi radialis (FCR), one of the prime mover muscles for the task we used. Excitability was also assessed on Day 11.

### Bimanual tracking task

**Figure 4:**
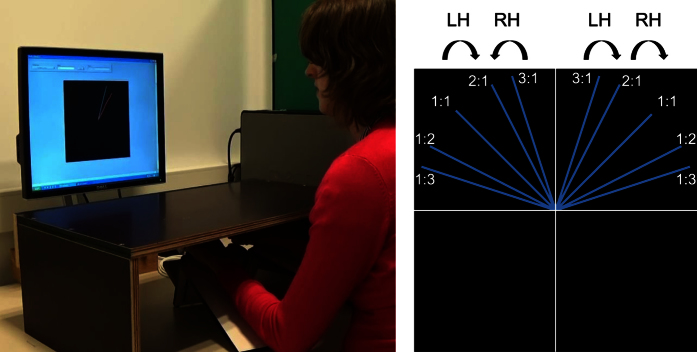
*B*imanual tracking task. The left panel shows the task set-up. Subjects were instructed to rotate two dials with both hands simultaneously in order to track the target dot that moves along the blue path on the screen. The trial shown here included delayed feedback (red line that showed the tracking trajectory at the end of the trial). Hands were hidden from view by a table-top bench. On the right panel, a schematic illustration of the frequency ratios with corresponding target blue paths is presented. The semi-circled arrows indicate the direction of movement for the left (LH) and the right (RH) hand, while the ratios indicate the required movement speed of the hands, i.e., the antecedent represents the speed of the left hand, while the consequent indicates the speed of the right hand.

Subjects performed a complex bimanual tracking task in which they had to track a moving dot by rotating two dials with both hands simultaneously. A general description of the task is given below, additional details can be found in Beets *et al*., and Sisti *et al*.^17^,^38^. The task combined skilled motor training in addition to cognitive control over coordination of both hands which requires intensive practice^17^,^38^. Starting from the centre of the display, a target dot moved along a straight line towards the edge of the display at different slopes (see Fig. 4). The left hand controlled movements of the cursor in the vertical direction, while the right hand controlled horizontal movements. Complexity of the task was increased by varying the relative frequency ratio (FR) of the hand movements. Different frequency ratios corresponded to different slopes of the target trajectory (see Fig. 4). A frequency ratio of 1:3 indicates the right hand (second digit in the ratio 1:3) had to move three times as fast as the left hand (first digit). Each trial consisted of two phases: a planning phase and a tracking phase. In the planning phase, the target trajectory was shown without the target moving. This allowed subjects to prepare their movements. After two seconds, the target dot started moving along the trajectory at a constant speed and subjects were instructed to track the dot (tracking phase, 7 seconds).

On every training day (Day 1–4), subjects underwent a pre-measurement, training, and a post-measurement. Pre- and post-measurements consisted of 2 trials per condition (5 FR × 2 quadrants × 2 trials = 20 trials) that were presented in one block and without feedback (only the target trajectory was visible, not the tracking trajectory). The training phase consisted of 10 trials per condition (5 FR × 2 quadrants × 10 trials = 100 trials). During training, conditions were presented in random order, a training scheme that has shown to be advantageous over blocked learning^37^,^60^. To optimize learning, we opted for fading feedback over training days. Participants either received concurrent feedback (showing the last second of the actual tracking trajectory along with the target trajectory), delayed feedback (static representation of the tracking trajectory was shown along with the target trajectory after the trial and not during tracking) or no feedback during the training trials. The number of trials with concurrent feedback was decreased during training and the number of trials without feedback was increased^61^,^62^. To keep participants motivated, a constant number of trials with delayed feedback was included in each training block. At retention (Day 11), a post-measurement of 4 trials per condition without feedback was completed (5 FR × 2 quadrants × 4 trials = 40 trials). Our outcome measure was the mean target deviation or the average Euclidian distance between target and position of the cursor at any point in time.

### Working memory tasks

To confirm previous findings on the beneficial effect of tDCS over DLPFC on working memory, we included two tasks in our protocol that measured different components of working memory in groups aDLPFC, sDLPFC, and BEHAV. First, to measure sustained attention, response inhibition and set-shifting, we applied a parametric Go/No-Go task^63^. Second, a traditional n-back working memory task was included in the protocol. Following previous studies^27^,^45^ and a pilot experiment, we have opted for a 3-back task to avoid ceiling and floor effects that might obscure subtle performance differences following tDCS. Detailed descriptions of the tasks are given as Supplementary Material.

### Questionnaires

At the start of each session, subjects reported the number of hours of sleep and the quality of sleep in the night proceeding the session on a 10-point scale. In addition, we asked for the number of units of alcohol and caffeine intake in the last 12 hours before the session.

After the BTT training with tDCS, the stimulation groups (aDLPFC, sDLPFC, aM1, and sM1) received a questionnaire about their perceived sensations during the stimulation. We asked to report the presence and severity of feelings of itchiness, tingling, headache, neck pain, scalp pain, burning, warmth/heat, pinching, iron taste, fatigue, concentration difficulties and acute mood changes. In addition, the onset and duration of these sensations was questioned and subjects were asked if the sensations influenced their performance.

### tDCS

tDCS was delivered by a battery-driven constant current stimulation (HDCStim class IIa; Model: HDCelEN-05, Newronika s.r.l., Milano, Italy). The common safety guidelines were followed^64^,^65^,^66^. The current was transferred by an anodal conductive-rubber electrode of 5 by 5 cm (current density: 0.08 mA/cm^2^) and a cathodal electrode of 6 by 8.5 cm (current density: 0.04 mA/cm^2^), which were placed in saline-soaked sponges. The choice for a bigger cathode was guided by the observation that increasing the size of the electrode turns the cathode in an effectively inactive electrode^55^. The centre of anodal electrode was placed over left M1 or left DLPFC. M1 was localized using a TMS hot spotting procedure. The left DLPFC, which corresponds to the F3 position in the international 10–20 system of EEG electrode placement, was determined by the method described in Beam, Borckardt, Reeves, and George^67^. In this method, the F3 position is calculated based on circumference, nasion-inion distance and the distance between the left and right pre-aurical point. The cathodal electrode was placed over the right supraorbital area at a minimal distance of 6 cm from the anodal electrode to decrease the probability of shunting of current through the scalp^68^. Electrodes were fixed to the head with a tubular elastic net bandage. In active conditions (aDLPFC and aM1), the stimulation lasted for 20 minutes at an intensity of 2 mA with a ramping up and down over 12 seconds. In the sham conditions (sDLPFC and sM1), the current turned off after 36 seconds in order to produce the same sensations as in the active conditions without the long-lasting effects of stimulation^69^. Subjects were blind to stimulation condition. Impedance was monitored by the stimulator.

### TMS

In aM1 and sM1, muscle activity was measured via EMG surface electrodes (Ag/AgCl) that were placed in belly-tendon montage over right FCR. A reference electrode was placed over the lateral epicondyle of the right arm. Raw signals were amplified with a gain of 1000 band-passed filtered (10–500 Hz) and sampled at 2000 Hz using a 16-bit analog to digital system (CED Limited, Cambridge, UK).

Single-pulse TMS was delivered with a standard figure of eight coil (70 mm loop diameter) connected to a Magstim 200 stimulator (Magstim Company, Dyfed, UK). The coil was positioned tangentially over scalp with the handle holding backward and laterally at 45° away from the mid-sagittal line. To maintain constant coil-positioning over the M1 hotspot of right FCR throughout the subsequent days of testing, the positioning and angles of the coil were monitored by a neuronavigation system (ANT, Enschede, The Netherlands). For navigation, a standard 3D anatomical MRI was used which was co-registered with the positions of the subject’s nasion, left ear, right ear and heap shape. After localization of the hotspot of the right FCR muscle in left M1, the position was marked with a semi-permanent marker for tDCS electrode placement. Subsequently, we measured resting motor threshold (rMT) on each day, prior to training and tDCS. The rMT is defined as the lowest intensity that elicits a motor evoked potential (MEP) of minimum peak-to-peak amplitude of 50 microvolts in 3 out of 5 consecutive trials. Before and after tDCS, we elicited MEPs by delivering 20 pulses at 130% of the individual rMT with an inter-stimulus interval of 5 seconds.

### Data analyses

BTT data were pre-processed in Labview (National Instruments) and MATLAB (Mathworks). A cluster analysis was performed in SPSS (IBM). All other statistical analyses were performed in R^70^. In case of violation of the sphericity assumption in ANOVA, a Greenhouse-Geisser correction was applied to the degrees of freedom and the p-value. Bonferonni correction for multiple comparisons was applied when necessary. The level of significance was set at p < 0.05. The data were also examined by estimating a Bayes factor using JZS Bayes factor repeated measures ANOVA with default prior scales, comparing the likelihood of the data under the null hypothesis and the alternative hypothesis^41^. For main effects, we have reported Bayes factors comparing the model including the relevant main effect and an effect of subjects (to account for the repeated measures) versus a null model with only an effect of subjects. For the interactions effects, the comparison between a model with the relevant interaction and an effect of subjects versus a null model with only an effect of subjects is meaningless if there is no evidence for including the main effects in the model. Therefore, Bayes factors for interaction effects are only reported when evidence for all main effects was observed. In that case, the reported Bayes factor compares a model with interaction, both main effects and an effect of subjects versus a null model that includes the main effects, the subject’s effect, but no interaction effect.

In the TMS data, trials in which root mean square (RMS) EMG exceeded 15 μV^71^ during the 40 ms immediately preceding the TMS pulse were discarded. Across subjects, 5.24% of the trials were excluded from analyses. In addition, we checked the stability of the baseline measures (pre-measurement on Day 1) by performing an unpaired t-test on the first and second half of the baseline data for each subject. After Bonferroni correction, no indication of an unstable baseline in any of the subjects was found. Subsequently, data were inspected for deviations from normality and averaged over all trials of a session. Next, data were normalized to an individual baseline by dividing the average MEP amplitude of each session by the average MEP amplitude of the first session (pre-measurement on Day 1).

## Additional Information

**How to cite this article**: Vancleef, K. *et al*. tDCS over left M1 or DLPFC does not improve learning of a bimanual coordination task. *Sci. Rep.* **6**, 35739; doi: 10.1038/srep35739 (2016).

## Supplementary Material

Supplementary Information
