# Supplementary material for: tDCS over left M1 or DLPFC does not improve learning of a bimanual coordination task
Source: Sci Rep. 2016 Oct 25;6:35739. doi: 10.1038/srep35739 (PMC5078840; doi:10.1038/srep35739)
Supplement: Supplementary Information [file srep35739-s1.pdf]

SUPPLEMENTARY MATERIALS TO

tDCS OVER LEFT M1 OR DLPFC DOES NOT IMPROVE LEARNING OF A BIMANUAL COORDINATION TASK

KATHLEEN VANCLEEF, RAF MEESEN, STEPHAN P. SWINNEN, HAKUEI FUJIYAMA

SUPPLEMENTARY METHODS: DESCRIPTION OF WORKING MEMORY TASKS

The Go/No-Go task [1] consisted of three difficulty levels in which black letters are presented for 500 ms with an inter stimulus interval of 100 ms on a white background of a computer monitor. In the first difficulty level, subjects had to respond as quickly as possible to the any of the target letters X, Y, or Z included in a stream of distractor letters (the other letters of the alphabet) each and every time they appear by pressing a key. This level only included go-responses and builds a prepotency for the target letters regardless of the context. Here, sustained attention was measured by the number of correct 'go' responses. In level 2, subjects had to respond to target letters X and Y (Z did not appear in the letter stream), but were required to inhibit a response to the last presented target. In other words, subjects responded to X and Y in alternation. Including a no-go-response that is context dependent increased the difficulty of the task. After each target letter, the working memory target set had to be shifted to the other target

letter. Because only two targets were included, keeping the current target in memory was sufficient. The number of correct no-go responses in this level gave an indication of response inhibition. In level 3, subjects again had to respond to the target and inhibit a response to the last presented target, but in this level, X, Y and Z were included in the letter stream. By adding an additional target letter, the ability to anticipate on the next response was decreased. Indeed, two target letters had to be kept in the working memory target set. This level substantially increased difficulty of the task and avoided ceiling effects that might occur in level two. Set shifting was assessed by the number of correct go-responses in level 3. Each level was tested twice in the following order: level 1, level 2, level 3, level 1, level 3, level 2. For each level, subjects first completed a practice block of 20 letters in which feedback was given. The practice block was followed by a letters stream of 100 letters that included 30 go trials for each level. At level 1, the 70 remaining letters were distractors. In level 2 and 3, 60 distractors and 10 no-go trials were included. This resulted in 25% no-go and 75% go trials in level 2 and 3 when distractors are discarded. The Go/No-Go task was programmed and presented using E-Prime (Psychology Software Tools).

In a 3-back task, series of four letters (X, C, R and M) were presented and subjects were asked to press a key if the letter shown was the same as the letter presented 3 stimuli before. The letters were presented for 2 seconds with an inter stimulus interval of 500 ms. The series of 48 letters were subdivided in three blocks of 16 letters and the occurrence of four target letters within each block was counterbalanced. Black letters were presented on a white background of a computer monitor by means of E-prime software (Psychology Software Tools). Correct responses, false positives and reaction time were measured. Reponses after the time frame of 2 seconds were registered as omissions.

SUPPLEMENTARY RESULTS: STATISTICAL VALUES FOR WORKING MEMORY TASKS

Table S.1 Statistical values for working memory tasks.

Effect	F	df_1	df_2	p	BF_{10}
Go/No-Go					
Sustained attention					
Day	10.66	1.85	77.83	< .001*	$5.48*10^{709}$
Group	0.96	2	42	.39	0.36
Group x Day	1.05	3.71	77.83	.39	na
Set shifting					
Day	44.68	1.88	79.08	< .001*	$1.25*10^{2256}$
Group	3.34	2	42	.05	1.71
Group x Day	0.24	3.77	79.08	.90	$9.25*10^{37}$
Response inhibition					
Day	1.09	1.99	83.62	.34	$2.07*10^{59}$
Group	0.06	2	42	.94	0.33
Group x Day	0.92	3.98	83.62	.46	na
3-back					
Percentage correct					
Day	0.56	1.98	83.01	.57	$4.75*10^{21}$
Group	0.18	2	42	.83	0.33
Group x Day	0.73	3.95	83.01	.57	na
Percentage false positives					
Day	5.08	1.45	60.87	.02*	$1.86*10^{407}$
Group	0.24	2	42	.79	0.34
Group x Day	0.32	2.9	60.87	.81	na
Reaction time					
Day	7.75	1;74	69.52	.002*	$7.40*10^{569}$
Group	1.06	2	40	.35	0.33
Group x Day	0.90	3.48	69.52	.46	na

* Refers to p-values below .05. ‘na’ indicates no Bayes Factor for the interaction effect was calculated because no evidence was observed to include both main effects in the model.

SUPPLEMENTARY RESULTS: DEMOGRAPHICS AND RESULTS OF QUESTIONNAIRES ON SLEEP, ALCOHOL, CAFFEINE AND TDCS SENSATIONS

Table S.2 Demographics and results of questionnaires on sleep, alcohol, caffeine and tDCS sensations

Dependent variable: Effect	F	df_1	df_2	p	BF_{10}
Age: Group	0.10	4	69	.98	0.06
Handedness coefficient: Group	0.80	4	70	.53	0.15
Sleep quality: Group	1.41	4	69	.24	0.12
Sleep duration: Group	1.31	4	69	.27	0.20
Alcohol intake: Group	0.72	4	69	.58	0.09
Caffeine intake: Group	0.47	4	69	.76	0.11
Severity of tDCS sensations					
Day	11.35	2.72	157.77	< .001*	1.60
Group (anodal vs sham)	0.14	1	58	.71	0.37
Day x Group	0.85	2.72	157.77	.46	na
Start of tDCS sensations					
Day	1.88	2.83	158.20	.14	$2.41*10^{221}$
Group (anodal vs sham)	5.36	1	56	.02*	2.91
Day x Group	0.88	2.83	158.20	.45	$1.41*10^{124}$
Duration of tDCS sensations					
Day	1.12	2.88	164.22	.34	$8.08*10^{157}$

Group (anodal vs sham)	2.4	1	57	.13	0.74
Day x Group	0.11	2.88	164.22	.95	na
Influence on performance of					
Day	1.07	2.84	164.49	.36	$3.33*10^{127}$
Group (anodal vs sham)	2.43	1	58	.12	0.78
Day x Group	0.46	2.84	164.49	.70	na

* Refers to p-values below .05. Handedness was measured with the Edinburgh Handedness Inventory[2] 'na' indicates no Bayes Factor for the interaction effect was calculated because no evidence was observed to include both main effects in the model.

1. Langenecker, S., Zubieta, J.-K., Young, E., Akil, H. & Nielson, K. A task to manipulate attentional load, set-shifting, and inhibitory control: Convergent validity and test–retest reliability of the Parametric Go/No-Go Test. *J. Clin. Exp. Neuropsychol.* **29,** 842–853 (2007).

2. Oldfield, R. The assessment and analysis of handedness: the Edinburgh inventory. *Neuropsychologia* **9,** 97–113 (1971).
